# Neural activity in mediodorsal nucleus of thalamus in rats performing a working memory task

**DOI:** 10.3389/fncir.2013.00128

**Published:** 2013-08-06

**Authors:** Jihyero Han, Ji Hyun Lee, Min Jung Kim, Min Whan Jung

**Affiliations:** ^1^Center for Synaptic Brain Dysfunctions, Institute for Basic Science, Korea Advanced Institute of Science and TechnologyDaejeon, South Korea; ^2^Neuroscience Laboratory, Institute for Medical Sciences, Ajou University School of MedicineSuwon, South Korea; ^3^Neuroscience Graduate Program, Institute for Medical Sciences, Ajou University School of MedicineSuwon, South Korea; ^4^Department of Biological Sciences, Korea Advanced Institute of Science and TechnologyDaejeon, South Korea

**Keywords:** prefrontal cortex, single unit, rat, delayed alternation, spatial working memory

## Abstract

The neural circuit consisting of mediodorsal nucleus (MDN) of thalamus and prefrontal cortex (PFC) has been implicated in working memory. In order to investigate whether and how the rodent MDN processes working memory-related signals, we recorded activity of single neurons from the MDN in rats performing a delayed spatial alternation task. The MDN conveyed significant neural signals for the animal's previously chosen goal (retrospective information) in the early delay period, but the signals deteriorated gradually over time so that they became weak toward the end of the delay period. Neural signals for the animal's upcoming goal choice (prospective information) were even weaker than those for the previously chosen goal. These results are in contrast to the finding in monkeys that both MDN and PFC persistently maintain task-related neural signals throughout delay period. Our results do not support sustained MDN-PFC interactions as a general mechanism for mediating working memory across different behavioral tasks and/or animal species.

## Introduction

Prefrontal cortex (PFC) is known to play an important role in working memory. Anatomically, one defining characteristic of the PFC is prominent afferent projections from the mediodorsal nucleus (MDN) of thalamus which receives strong return projections back from the PFC (Fuster, 2008). Previous studies in primates have suggested that dorsolateral PFC (DLPFC)-MDN circuit may play an important role in working memory. First, results from lesion/inactivation studies indicate requirement of both structures for working memory. In monkeys, damaging, or inactivating either DLPFC (Jacobsen, 1935; Pribram et al., 1952; Olds, 1966; Fuster and Alexander, 1970 or MDN (Schulman, 1964; Olds, 1966; Isseroff et al., 1982; Aggleton and Mishkin, 1983a,b; Zola-Morgan and Squire, 1985; Parker et al., 1997) impairs the animal's performance in a variety of working memory tasks. In humans, damages in the MDN often induce behavioral deficits similar to those observed after damages in the PFC (“prefrontal syndromes”), disrupting executive functions that require working memory (Joyce and Robbins, 1991; Daum and Ackermann, 1994; Van Der Werf et al., 2000, 2003; Zoppelt et al., 2003). Second, brain imaging studies in humans have found increased blood oxygenation level-dependent signals in the DLPFC (Owen et al., 2005) as well as MDN (Elliott and Dolan, 1999; De Zubicaray et al., 2001) during working memory tasks. Third, physiological studies in monkeys have shown that some neurons in the DLPFC (reviewed in Funahashi and Kubota, 1994; Fuster, 2008) as well as MDN (Fuster and Alexander, 1971, 1973; Tanibuchi and Goldman-Rakic, 2003; Watanabe and Funahashi, 2004a,b) show persistent activity during delay period with their activity tuned to sensory features of cues/movement directions of the animals. These results show that both the DLPFC and MDN maintain information required for correctly performing a working memory task throughout delay period. Finally, cooling the DLPFC impaired performance of monkeys in a delayed response task and concomitantly disrupted sustained elevation of MDN neural activity during delay period (Alexander and Fuster, 1973), suggesting functional interdependence between the two structures.

Consistent results have been obtained from behavioral studies in rodents. Numerous studies in rats have shown that lesions/inactivation of the medial wall of the PFC (mPFC) (reviewed in Kolb, 1984; Brown and Bowman, 2002; Uylings et al., 2003; Vertes, 2006; Seamans et al., 2008; Kesner and Churchwell, 2011) or MDN (e.g., Winocur, 1985; Stokes and Best, 1990; Harrison and Mair, 1996; Floresco et al., 1999; Romanides et al., 1999) lead to impaired performance in various working memory tasks. Physiological studies also have shown that mPFC neurons in rats carry task-related information during various working memory tasks (Sakurai and Sugimoto, 1986; Orlov et al., 1988; Batuev et al., 1990; Chang et al., 2002; Fujisawa et al., 2008). We also have shown that rat mPFC neuronal population robustly transmitted information about the animal's past/future goal choices throughout delay period in a spatial delayed alternation task (Baeg et al., 2003). However, to our knowledge, few studies have examined working memory-related neuronal activity in the rodent MDN (c.f. Sakurai and Sugimoto, 1986), which was the subject of the present study. In order to investigate whether and how the rat MDN processes working memory-related signals, we recorded single unit signals from the MDN while rats were performing a delayed spatial alternation task.

## Materials and methods

### Subjects

Fifteen young (12–16 weeks old, 300–320 g) male Sprague-Dawley rats were used in the present study. The animals were individually housed in a colony room and initially allowed free access to food and water. They were then handled extensively while adapting to water deprivation for 1 week, and, once behavioral training began, restricted to 30 min of access to water after finishing one behavioral session per day. Their body weights were maintained at >80% *ad libitum* body weights throughout the experiments. Behavioral testing was performed in the dark phase of a 12-h light/dark cycle. The experimental protocol was approved by the Ethics Review Committee for Animal Experimentation of the Ajou University School of Medicine, Korea.

### Behavioral task

**Figure 1:**
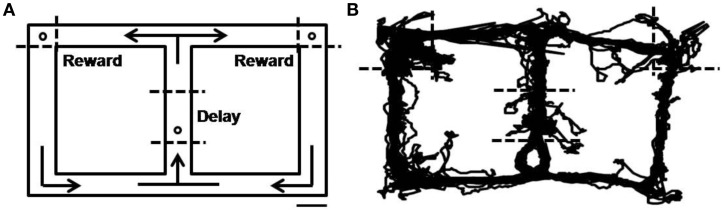
**Behavioral task. (A)** Starting from the central stem, rats were required to visit two goal sites (open circles on the upper left and upper right corners) alternately to obtain water reward (20 μl). They were required to come back from the reward sites to the central stem via the lateral alley. The animals were also rewarded with water (20 μl) on the central stem (open circle) in all trials. Dashed lines denote approximate boundaries for the delay and reward periods, and arrows indicate movement directions. Scale bar, 10 cm. **(B)** An example of rat's movement trajectory in one behavioral session.

The behavioral task was a delayed spatial alternation task on a figure eight-shaped maze (Jung et al., 1998; Baeg et al., 2003). The dimension of the maze was 100 × 70 cm and it was elevated 25 cm from the floor. The track was 9 cm in width with 2.5 cm walls along the entire track (Figure 1). The animals had to alternate between two goal sites (upper left and upper right corners of the maze; Figure 1) where water reward (20 μl) was delivered in correct trials. No sensory cue was provided to the animals in this task so that they had to choose a goal in each trial based on which goal they had visited in the previous trial. Consecutive visits to the same goal site was regarded as an error and not rewarded. The animals were trained to come back to the central stem via the lateral alley after visiting a goal site (see arrows in Figure 1). They were rewarded with water (20 μl) on the central stem (Figure 1) in all trials, and their stay on the central stem served as the delay period that varied trial to trial. The animals were over-trained in the task (12–14 days, 33 ± 8 trials per day; mean ± *SD*) before surgery, and re-trained on the task for 5 days before unit recording, so that they did not navigate along invalid paths (e.g., directly going from one goal site to the other) during recording sessions. Water was delivered through a solenoid valve and its delivery was controlled by a personal computer using LabView software (National Instruments).

### Unit recording

**Figure 2:**
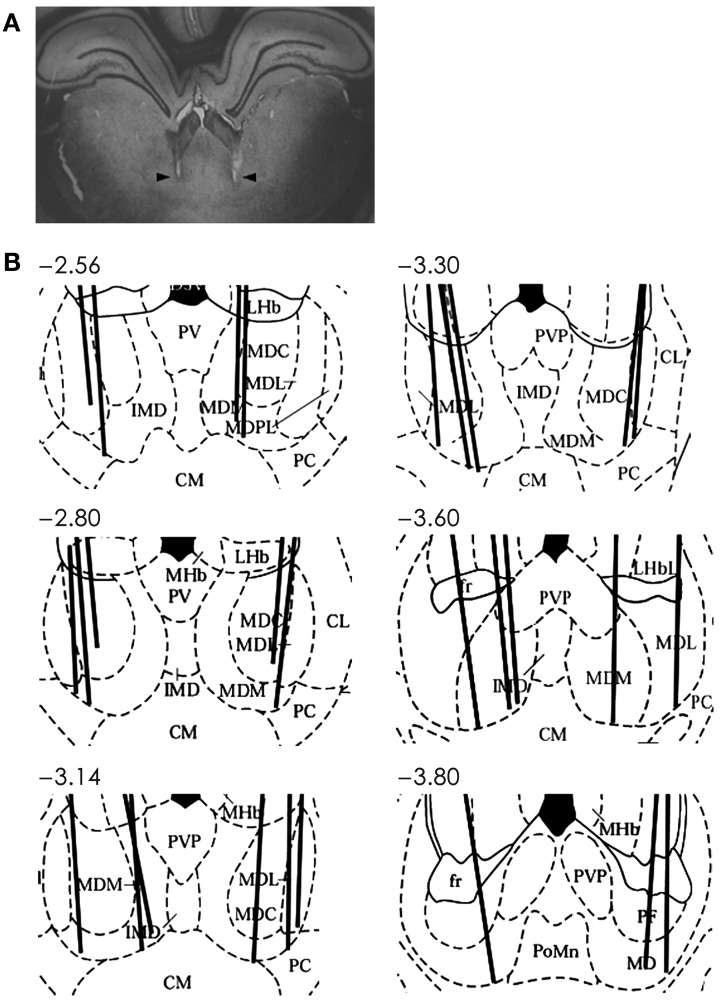
**Recording sites in the MDN. (A)** An example photomicrograph (coronal section; approximately 3.6 mm posterior to bregma) showing two electrode tracks (arrows). **(B)** Reconstruction of electrode tracks. Each solid line denotes one tetrode track. Numbers indicate distances (mm) from bregma. AD, anterodorsal thalamic nucleus; CM, centralmedial thalamic nucleus; CL, centrolateral thalamic nucleus; D3V, dorsal third ventricle; IMD, intermediodorsal thalamic nucleus; LHb, lateral habenular nucleus; LHbL, lateral habenular nucleus, lateral; LHbM, lateral habenular nucleus, medial; MD, mediodorsal nucleus; MDC, central segment of mediodorsal nucleus; MDL, lateral segment of mediodorsal nucleus; MDM, medial segment of mediodorsal nucleus; MDPL, paralaminar segment of mediodorsal thalamic nucleus; MHb, medial habenular nucleus; PC, paracentral thalamic nucleus; PT, paratenial thalamic nucleus; PV, paraventricular thalamic nucleus; PVA, paraventricular thalamic nucleus, anterior; PVP, paraventricular thalamic nucleus, posterior. Modified with permission from Elsevier (Paxinos and Watson, 1998).

Single units were recorded as described in our previous study (Baeg et al., 2001). Briefly, two tetrodes, each attached to a microelectrode drive (McNaughton et al., 1989), were implanted in two hemispheres with 5° angles toward the midline aiming the MDN (2.5–3.8 mm posterior, 1.2 mm lateral from bregma and 4.8–6.0 mm ventral from the brain surface; Figure 2) under deep sodium pentobarbital anesthesia. Upon recovery from surgery, tetrodes were gradually advanced to obtain unit signals from the intended recording sites. Unit signals were recorded from both tetrodes simultaneously via an FET source-follower headstage mounted on the animal's head, band-pass filtered between 0.6–6 KHz, amplified 10,000×, digitized at 32 KHz, and stored on a SUN 4u workstation using a Cheetah data acquisition system (Bozemann, MT, USA). The animal's head position was also recorded by tracking two sets of infrared LEDs mounted on the headstage at 20 Hz. Upon completion of recordings, an electrolytic current (50 μA, cathodal, 30 s) was applied through one channel of each tetrode to generate marking lesions. Recording locations were verified based on light microscopic examinations of electrode tracks and marking lesions as previously described (Baeg et al., 2001).

### Analysis

#### Behavioral stages

The delay period was determined separately for each behavioral session based on the animal's movement trajectories that were monitored by tracking the animal's head position. The onset and offset of the delay period were determined as the first and last time points on the central stem during which the animal's lateral head position did not vary significantly (temporal resolution, 50 ms; *t*-test, alpha = 0.05) depending on the animal's previous and upcoming goal choice, respectively (c.f., Kim et al., 2009; Sul et al., 2010, 2011). The onset of the reward period was when the animal arrived at either goal location (4.5 cm away from each goal) starting from the central stem and its end was when the animal left the goal area (4.5 cm below each goal). Mean (± *SD*) durations of the delay and reward periods were 3.9 ± 3.8 and 4.3 ± 2.0 s, respectively.

#### Unit isolation

**Figure 3:**
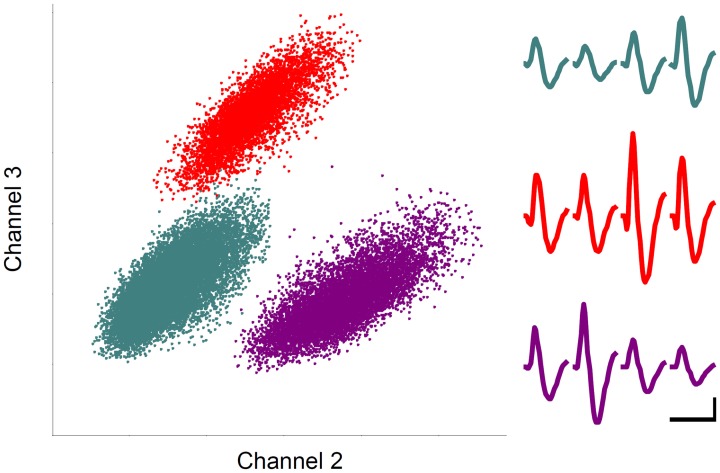
**Unit recording example.** Each unit cluster is indicated in different color. Horizontal and vertical axes indicate the amplitudes of spike signals recorded through two different channels of a tetrode. Averaged spike waveforms recorded through four tetrode channels are shown in corresponding colors on the right. Calibration: 1 ms and 0.1 mV.

Recorded unit signals were processed off-line. Putative single units were isolated by manually drawing boundaries on two dimensional projections of various spike waveform parameters (peak amplitude and width of filtered spike waveforms; Figure 3) as previously described (Baeg et al., 2001).

#### Bias index

As an index for modulation for each neuron of MDN delay-period activity by the animal's previous or upcoming goal choice, mean discharge rates during the entire delay period were averaged across trials according to the trial type (left vs. right goal choice) as the following:

Index=(A−B)/(A+B),

where A and B denote averaged delay-period activity for the animal's left and right goal choices, respectively. The index was calculated separately for the previous and upcoming goal choice.

#### Goal choice decoding

We examined how well individual neuronal activity during the delay period predicted the animal's previous or upcoming goal choice using a Bayesian decoding procedure (Baeg et al., 2003) as well as a discriminant analysis (Duda et al., 2001) with leave-one-out cross-validation. A single trial was removed, and the animal's previous or upcoming goal choice for that trial was predicted based on neural activity and the animal's goal choices in the remaining trials. This procedure was repeated for all trials and the percentage of predictions that matched the animal's actual choices (% correct decoding) was calculated.

#### Regression analysis

The decoding analyses do not take the animal's movement into consideration. In order to examine delay-period neural activity correlated with the animal's previous or upcoming goal choice while controlling for the animal's positional variation during the delay period, we ran a multiple linear regression analysis that included the animal's previous and upcoming goal choices as well as the animal's lateral head position as explanatory variables. In this analysis, trial-by-trial neuronal activity during successive 1-s long time windows of the delay period (that were advanced by 50 ms) was related to trial-by-trial choices of the animal using the following regression model:

(1) $$S\left(i\right)=a_{0}+a_{1}C\left(i-1\right)+a_{2}C\left(i\right)+a_{3}P\left(i\right)+\varepsilon ,$$

where *S(i)* indicates average spike discharge rate within a given analysis time window in trial *i*, *P(i)* is the animal's mean lateral head position during the analysis time window in trial *i* (ranged from 115 to 239 pixels), *C(i)* is the animal's goal choice in trial *i* (i.e., upcoming goal choice; dummy variable, 1 and –1), *C(i–1)* is the animal's goal choice in trial *i–1* (i.e., previous goal choice; dummy variable, 1 and –1), ε is the error term, and *a*_0_~ *a*_3_ are regression coefficients.

We additionally ran the following two regression models that contained either the previous or upcoming goal choice, but not both, as an explanatory variable in order to apply a more liberal criterion for neural activity correlated with the previous or upcoming choice, and also to determine whether neural activity was more correlated (in terms of *R*^2^ value) with the previous or upcoming choice:

(2) $$S\left(i\right)=a_{0}+a_{1}C\left(i-1\right)+a_{2}P\left(i\right)+\varepsilon ,$$

(3) $$S\left(i\right)=a_{0}+a_{1}C\left(i\right)+a_{2}P\left(i\right)+\varepsilon .$$

#### Continuous differential delay cells

We examined how many MDN neurons conveyed signals for the previous goal choice throughout the delay period. For comparison with our previous results in the mPFC (Baeg et al., 2003), we defined “continuous differential delay cells” as in our previous study. For each neuron, all trials in a given session were divided into two groups according to the animal's previous goal choice (left vs. right) and average firing rate over the whole delay period was calculated for each group. If average firing rates of the two groups were significantly (*t*-test, *p* < 0.01) different from each other, the neuron was considered as a differential delay cell. For each differential delay cell, the entire delay period was divided into four equal-duration bins and mean firing rate in each bin was calculated using the trial group with a higher mean firing rate. If mean firing rates in all four bins were higher than the mean firing rate during the time period other than the delay period (*t*-test, *p* < 0.05 uncorrected for multiple comparisons), the unit was considered as a continuous differential delay cells.

#### Statistical analysis

A *p* value < 0.05 was used as the criterion for a significant statistical difference unless noted otherwise. Significant difference of goal-choice decoding from chance level (50% correct prediction) and significance of a regression coefficient were tested based on a *t*-test. A binomial test was used to test whether the probability to obtain a given or higher proportion of significant (goal-choice decoding or a regression coefficient) neurons is less than 5%. Significance of activity difference between two goal locations (left vs. right) and two choice outcomes (correct vs. error) was tested with Wilcoxon rank-sum tests.

## Results

### Neuronal database

Single units were recorded from the MDN while 15 rats were performing 12–14 daily sessions each consisting of 18–63 trials (33 ± 8, mean ± *SD*) of the delayed spatial alternation task (Figure 1). A total 92 well-isolated single units were recorded and their overall mean (± *SD*) discharge rate during the entire recording session was 11.2 ± 12.3 (0.9–78.9) Hz. The units were recorded from all segments of the MDN (Figure 2) and we found no evidence for regional specialization of neurons encoding particular variables of interest. Up to three well-isolated units were simultaneously recorded from one tetrode (Figure 3), but typically only one well-isolated unit was recorded from one tetrode (mean ± *SD* = 1.3 ± 0.6 units per tetrode). Also, unit signals were recorded from only one tetrode except in one session. Therefore, the number of simultaneously recorded units per session (from two tetrodes) was small (1–3, mean ± *SD* = 1.3 ± 0.6 units per session).

### Neural activity during delay-period

**Figure 4:**
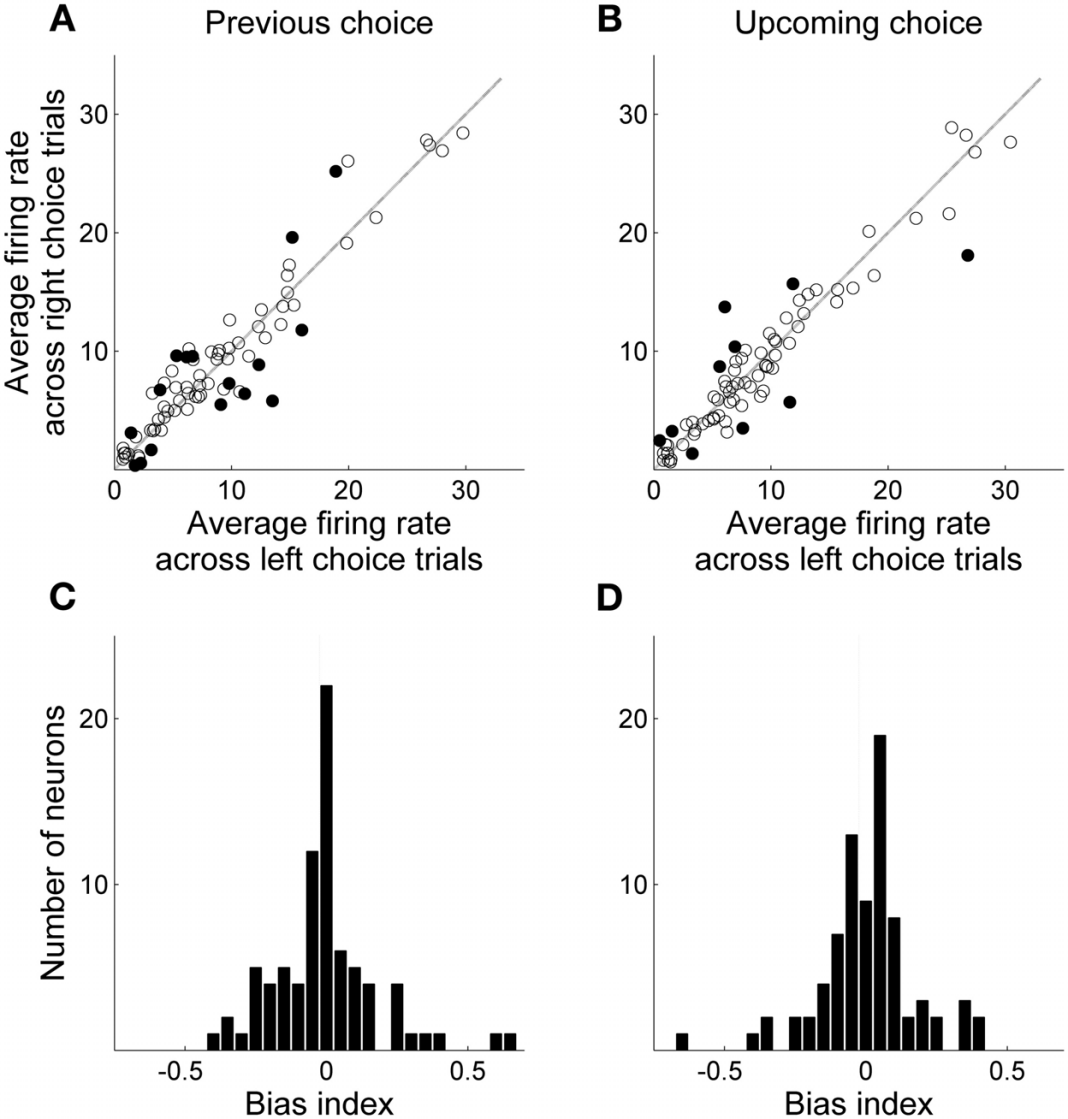
**Mean delay-period activity of MDN units associated with left and right goal choices.** All trials were divided into two groups according to the animal's previous (**A** and **C**) or upcoming (**B** and **D**) goal choice and mean discharge rates during the entire delay period were averaged across trials within each group for each neuron. **(A,B)** Scatter plots relating averaged delay-period activity of individual neurons for the left and right goal choices. Filled circles denote those neurons for which average firing rates during the delay period were significantly different between left and right choices (*t*-test, alpha = 0.05). **(C,D)** Frequency histograms for the bias index.

**Figure 5:**
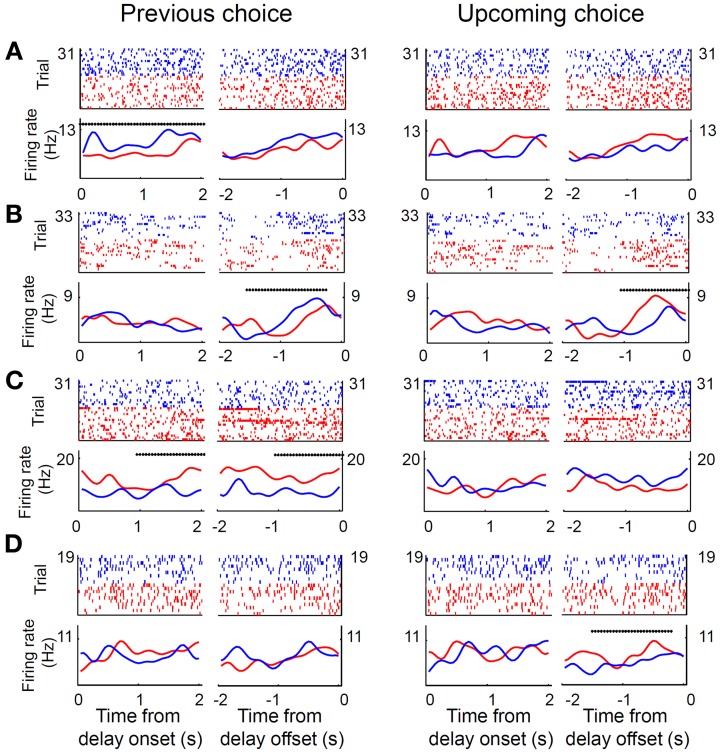
**Examples of MDN neural activity during delay period. (A–D)** Shown are four unit examples. Trials were divided into two groups according to the previous (left panel) or upcoming (right panel) goal choice (red, left goal choice; blue, right goal choice). Top, spike raster plots. Each tick mark indicates a spike and each row is one trial. Each trial was aligned to the onset or offset of the delay period. Bottom, spike density functions that were generated by applying a Gaussian kernel (σ = 100 ms) to the corresponding spike trains in the raster plots. Asterisks denote significant dependence of unit activity on the animal's previous (left panel) or upcoming (right panel) goal choice (Equation 1) during a 1-s time window (asterisk aligned at the center of the window) that was advanced in 50-ms time steps.

Only those sessions with at least one error trial (60 sessions, 80 neurons) were included in the analysis of delay-period activity, unless noted otherwise, in order to compare neural activity related to the previous and upcoming goal choices. Mean discharge rates of the 80 units during the entire delay period ranged between 0.8 and 90 Hz (overall mean ± *SD* = 11.6 ± 13.4). Their delay-period activity was modulated by the animal's previous (or upcoming) goal choice in different degrees and in different directions. Some neurons showed higher discharge rates during the delay period after (or before) the left goal choice, whereas other units showed higher discharge rates after (or before) the right goal choice (Figure 4). Moreover, dependence of delay-period activity on the animal's previous or upcoming goal choice was not static throughout the delay period, but dynamically changed in the course of the delay period. Examples of MDN units that showed different firing according to the animal's previous or upcoming goal choice during certain portions of the delay period are shown in Figure 5.

To examine how much information individual units carried about the animal's previous or upcoming goal choice, we predicted the animal's trial-by-trial goal choices based on delay-period activity (mean discharge rate in each trial) of individual neurons using a Bayesian decoding procedure (Baeg et al., 2003) as well as a discriminant analysis (Duda et al., 2001). In the Bayesian decoding, delay-period activity of MDN units poorly predicted the animal's actual choices (mean ± SEM, previous goal choice, 51.1 ± 1.4% correct prediction; upcoming goal choice, 50.3 ± 1.5%; comparison with chance level (50%), *t-test*, previous goal choice, *p* = 0.423, upcoming goal choice, *p* = 0.815). A discriminant analysis yielded somewhat better predictions that were significantly above chance level (previous goal choice, 55.1 ± 1.5% correct prediction; *t*-test, *p* = 0.001; upcoming goal choice, 55.0 ± 1.4%; *p* = 0.001), but they were substantially lower compared to the predictions by mPFC neurons (Bayesian decoding, >60% correct prediction on average; Baeg et al., 2003). These results indicate that delay-period activity of individual MDN neurons carried lower information about the animal's goal choices than mPFC neurons.

**Figure 6:**
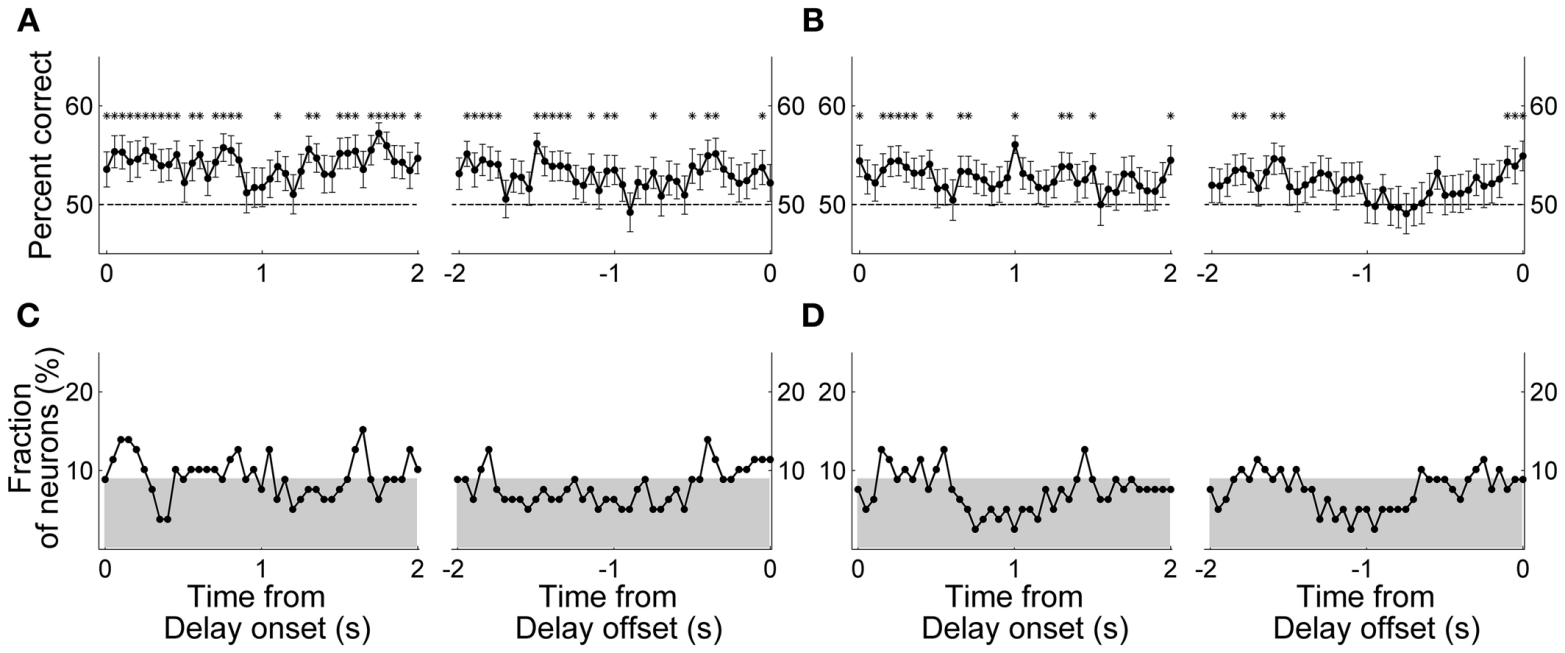
**Neural decoding of the animal's goal choice.** The animal's trial-by-trial goal choices (**A** and **C**, previous goal choice; **B** and **D**, upcoming goal choice) were predicted using a discriminant analysis based on individual neuronal activity during a 1-s time window that was advanced in 50-ms steps. In each panel, the first data point of the left graph (time from delay onset) corresponds to the first 1 s of the delay period, and the last data point of the right graph (time from delay offset) corresponds to the last 1 s of the delay period. **(A,B)** Percentages of correct prediction averaged across all analyzed neurons (*n* = 80). Asterisks denote significant difference from chance level (50%; *t*-test, *p* < 0.05). The error bars are SEM. **(C,D)** Fractions of neurons with significant decoding of the animal's goal choice. The shading indicates chance level (binomial test, alpha = 0.05).

Next, in order to examine the time course of MDN delay-period activity related to the animal's previous and upcoming goal choices, we repeated the same analyses using a 1-s time window that was advanced in 50-ms time steps. We aligned the beginning of the first analysis window to the onset of the delay period (alignment to delay onset), and also aligned the end of the last analysis window to the offset of the delay period (alignment to delay offset). Trials with the duration of the delay period <1 s (0.9 ± 2.3 trials per session; mean ± *SD*) were excluded from this and subsequent sliding window analyses. The results were similar to those obtained with the analysis using the entire delay-period activity. In the Bayesian decoding, the percentage of correct decoding was near 50% and insignificant for most of the analysis windows (data not shown). In the discriminant analysis, the percentage of correct decoding was slightly higher than 50% and significant in many analysis windows (Figures 6A,B). However, when we counted the fraction of individual MDN neurons with significant decoding based on the discriminant analysis (i.e., significant difference from 50%; *t*-test, *p* < 0.05), the fraction fluctuated around the chance level (binomial test, alpha = 0.05; Figures 6C,D).

**Figure 7:**
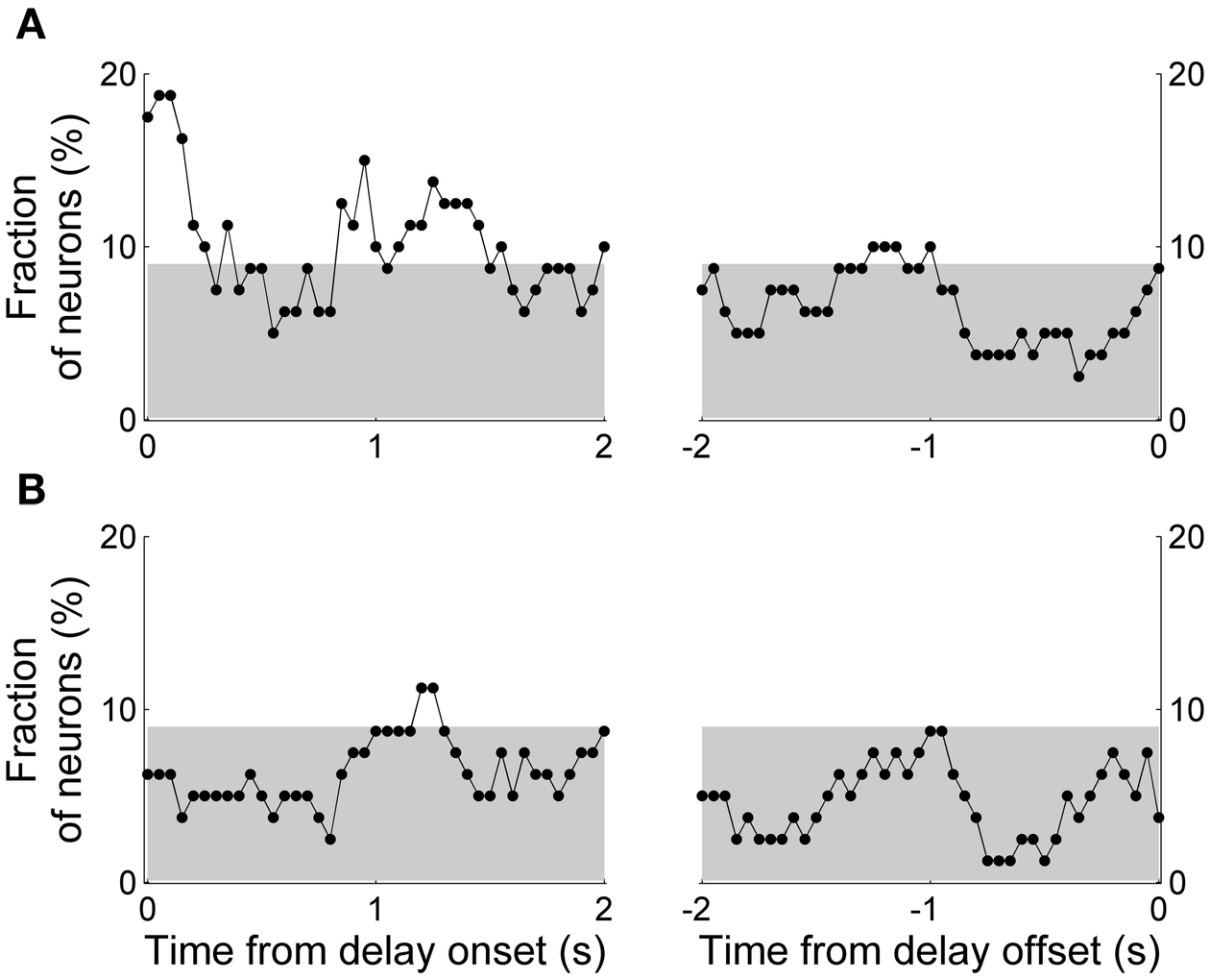
**Results of regression analysis.** The graphs show fractions of neurons with significant dependence of their activity on the animal's previous **(A)** or upcoming **(B)** goal choice (Equation 1) during a 1-s time window that was advanced in 50-ms steps. As in Figure 6, the first data point of the left graph (time from delay onset) corresponds to the first 1 s of the delay period, and the last data point of the right graph (time from delay offset) corresponds to the last 1 s of the delay period. The gray shading indicates the chance level (binomial test, alpha = 0.05).

The above decoding analyses do not take into account movement variations during delay period that can potentially affect neural activity (Euston and McNaughton, 2006; Cowen and McNaughton, 2007). We therefore performed a multiple regression analysis that examined dependence of delay-period activity on the animal's previous and upcoming goal choices considering variations of the animal's lateral head position during the delay period (Equation 1; activity from example neurons shown in Figure 5). We quantified the fraction of neurons with significant dependence of their activity on the animal's previous or upcoming goal choice during a 1-s sliding window that was advanced in 50-ms time steps. Again, only those sessions with at least one error trial were included in the analysis (60 sessions, 80 neurons), and trials with delay duration <1 s were excluded. The results showed that the strength of choice signals (i.e., fraction of neurons whose activity significantly depended on the animal's previous or upcoming goal choice) was weak; significant (binomial test, *p* < 0.05) fractions of neurons showed dependence of their activity on the animal's previous goal choice mostly during the early delay period, and the fraction of neurons showing dependence of their activity on the animal's upcoming goal choice was below chance level (binomial test, *p* < 0.05) for the large part of the delay period (Figure 7).

**Figure 8:**
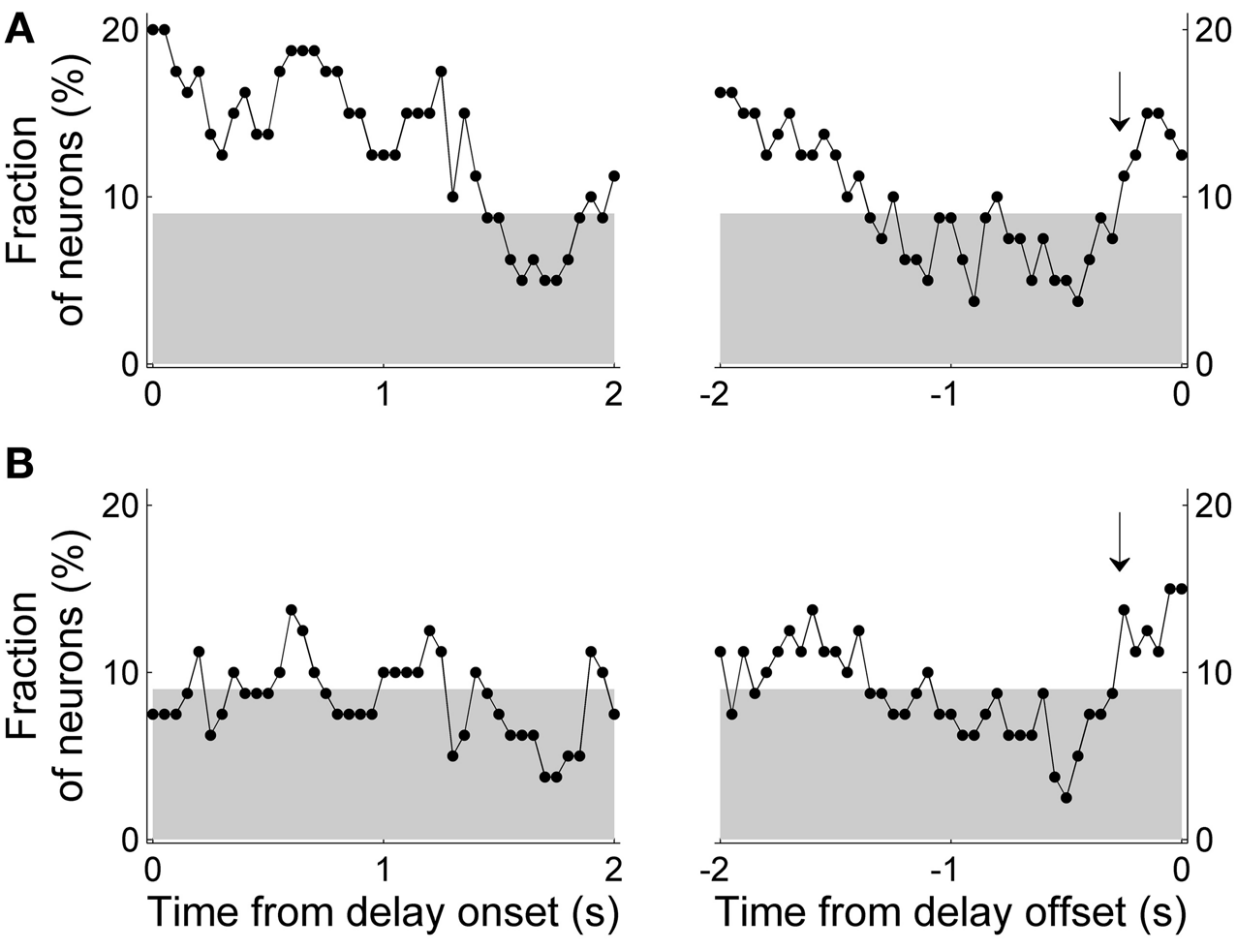
**Results of regression analysis with a relaxed criterion.** Either the previous **(A)** or upcoming **(B)** goal choice (instead of both) was included in the regression model (Equations 2 and 3, respectively). Same format as in Figure 7.

The above results suggest that MDN neural activity related to the animal's previous and upcoming goal choice is not persistently maintained throughout the delay period and that upcoming choice-related neural activity is weaker than previous choice-related neural activity. We performed several additional analyses to confirm these observations. First, we relaxed the selection criterion for those neurons that changed their activity according to the animal's previous or upcoming goal choice. We included only the previous or upcoming goal choice instead of both terms in the regression model (Equations 2 and 3) so that there was no potential multicolinearity problem and neural activity related to one term (previous or upcoming choice) might be captured to be related to the other. Significant (binomial test, *p* < 0.05) numbers of neurons showed dependence of their activity on the animal's previous goal choice approximately for the initial 1.5 s of the delay period. The fraction of neurons signaling the previous goal choice gradually decreased over time so that it became insignificant during the later phase of the delay period (Figure 8A). When the analysis window was aligned to the delay offset, the fraction of neurons signaling the previous goal choice became insignificant ~1.5 s before the delay offset, although previous choice signals arose above chance level before delay offset (Figure 8A, arrowhead). A somewhat different pattern was observed for neural signals for the upcoming goal choice. Neural signals for the upcoming goal choice fluctuated around chance level (binomial test, alpha = 0.05) for the most part of the delay period. The upcoming choice signals arose above chance level before delay offset, however, similar to the previous choice signals (Figure 8B, arrowhead). Thus, persistent signals for the previous or upcoming goal choice were not observed throughout the delay period even with the relaxed criterion and signals for the previous goal choice were stronger than those for the upcoming goal choice especially during the early delay period.

Second, we examined how many MDN units persistently maintained their activity throughout the delay period. We first examined how many neurons signaled the animal's goal choice in the first as well as the last 1 s of the delay period. The neurons that signaled the previous or upcoming goal choice (determined with Equations 2 and 3, respectively) during the first 1 s of the delay period did not overlap much with those during the last 1 s of the delay period (Table 1). We also determined the number of “continuous differential delay cells” that were defined as in our previous study in the mPFC (Baeg et al., 2003). For comparison with our previous results, all recorded neurons from all sessions were subject to this analysis (instead of only those sessions with at least one error trial). Only nine out of 92 (9.8%) met the criteria for continuous differential delay cells. These results further indicate that only a small fraction of MDN neurons conveyed signals for the animal's goal choice based on sustained activity throughout the delay period.

**Table 1:** **Distribution of units signaling the previous and upcoming goal choices**.

	**First 1 s**	**Last 1 s**	**Both periods**
PG	16 (20%)	10 (12.5%)	3 (3.8%)
UG	6 (7.5%)	12 (15%)	1 (1.3%)
Both choices	5 (6.3%)	7 (8.6%)

Shown are the numbers (and proportions) of units signaling the previous and upcoming goal choices (PG and UG, respectively) during the first and the last 1 s of the delay period. Both choices, those units signaling PG as well as UG during a given analysis time window. Both periods, those units signaling the animal's goal choice (PG or UG) during the first as well as the last 1 s of the delay period.

**Figure 9:**
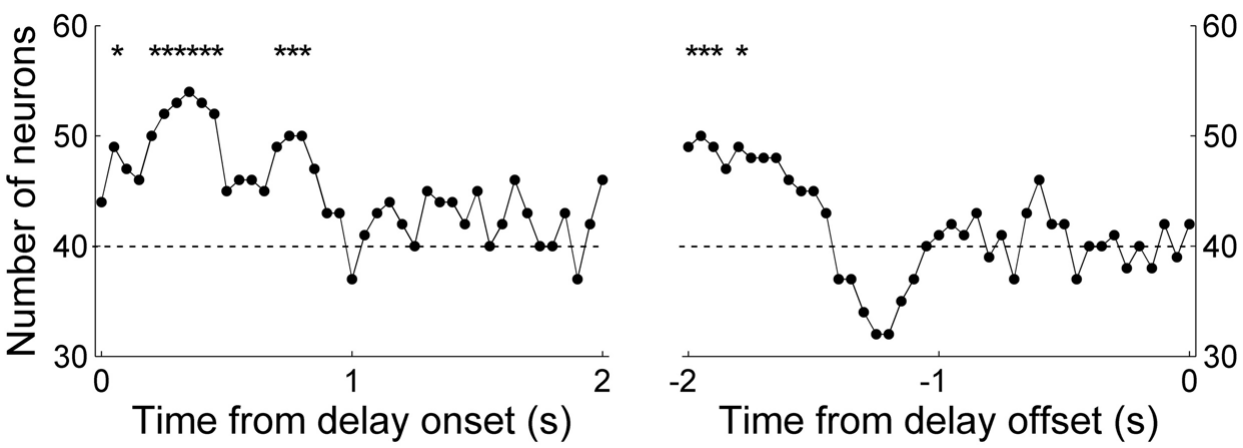
**Comparison of neural activity related to the previous and upcoming goal choices.** The graphs show the number of neurons whose activity is better explained by the animal's previous than upcoming goal choice (Equation 2 vs. Equation 3; 1-s window advanced in 50-ms steps). The dashed horizontal line indicates equal distribution (50%). The first data point of the left graph (time from delay onset) corresponds to the first 1 s of the delay period, and the last data point of the right graph (time from delay offset) corresponds to the last 1 s of the delay period. Asterisks indicate significant difference from chance level (χ^2^-test, *p* < 0.05).

We also examined relative strengths of the previous and upcoming choice signals by determining the number of neurons whose activity was better explained by the previous than upcoming goal choice (in terms of *R*^2^ values of Equations 2 and 3) in a 1-s sliding window that was advanced in 50-ms time steps. The number of neurons whose activity was better explained by the previous than upcoming goal choice was significantly larger than expected by chance (χ^2^-test, *p* < 0.05) in several analysis windows especially during early delay period, whereas the opposite (i.e., significantly larger number of neurons whose activity was better explained by upcoming goal choice) was not observed in any analysis window (Figure 9). These results are consistent with the results of the other analyses indicating preferential encoding of the animal's previous goal choice (retrospective information) by MDN neurons during early phase of the delay period.

### Neural activity during reward period

**Figure 10:**
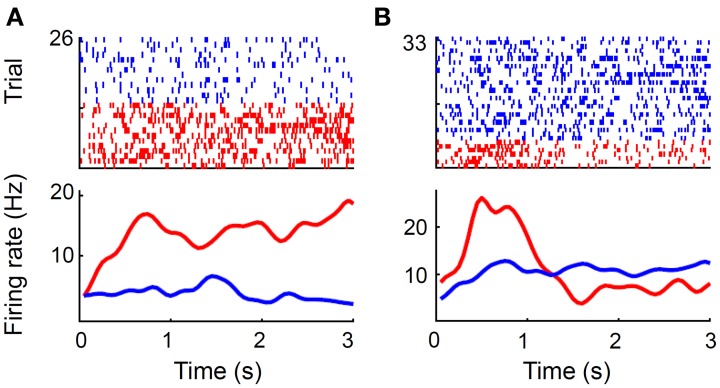
**Reward-related neural activity. (A)** A unit that changed its firing rate during the reward period according to goal location. Trials were divided according to the chosen goal (red, left; blue, right). Only correct trials are shown. **(B)** A unit that changed its firing rate according to trial outcome. Trials were divided according to trial outcome (red, error; blue, correct). A spike raster plot (top) and a spike density function (σ = 100 ms) are shown for each example. Time 0 indicates the onset of the reward period.

Thirty-six out of 92 (39.1%) MDN neurons (all 72 sessions were analyzed) showed significantly different firing (Wilcoxon rank-sum test, *p* < 0.05) during the first 1 s of the reward period according to goal location, which is significantly above chance level (binomial test, *p* << 0.001; Figure 10A). Similar results were obtained when we analyzed mean neural activity during the entire reward period (42 out of 92 neurons, 45.6%; *p* << 0.001). We analyzed only those sessions with at least three error trials (32 sessions, 44 neurons) when examining neural activity related to choice outcome (correct vs. error) as well. During the first 1 s of the reward period, 17 (38.6%) and seven (15.9%) neurons showed significantly different firing according to goal location and choice outcome (Figure 10B), respectively, and five of them showed significantly different firing according to both goal location and choice outcome. There was a trend for choice-outcome encoding neurons to encode reward location as well (Fisher's exact test, *p* = 0.057). These results show that the MDN conveyed concurrent neural signals for choice outcome and goal location during the reward period.

## Discussion

In order to obtain insights on the role of PFC-MDN interactions in working memory, we recorded MDN unit activity in rats performing a spatial delayed alternation task. We have shown previously that rat mPFC neurons convey retrospective/prospective information throughout delay period in the same behavioral task (Baeg et al., 2003). In that study, we recorded mPFC unit activity both during and after training to keep track of changes in neural activity in the course of learning a new working memory task. In the present study, we recorded MDN unit activity only in well-trained animals (i.e., after training) to examine whether and how MDN units transmit trial-by-trial working memory-related signals, which has been largely unknown. In a rare physiological study in rats performing a delayed go/no-go alternation task (Sakurai and Sugimoto, 1986), MDN neural activity depended significantly on the response type (go vs. no-go) in the early as well as late half (5 s each) of the delay period, suggesting that MDN neurons carried retrospective/prospective information required to perform the task throughout the delay period. However, this study was based on multi-unit, rather than single-unit recordings, and potential influence of behavioral variations during the delay period was not addressed. In the present study, we recorded single unit activity in the MDN, and analyzed delay-period neural activity taking into account variations in the animal's head position. Employing these approaches, we found that, unlike in monkeys, retrospective/prospective neural signals in rats were not persistently maintained throughout delay period. The MDN conveyed stronger retrospective than prospective neural signals, especially during early delay period, but even these retrospective neural signals gradually decayed over time so that they were below chance level toward the end of the delay period. Our results indicate that the MDN does not persistently maintain working memory-related neural signals throughout delay period in all working memory tasks and/or in all animal species.

Sustained neural activity in the PFC during delay period is a strong candidate for neural substrate of working memory (Funahashi and Kubota, 1994; Fuster, 2008), and it has long been suggested that sustained delay-period neural activity might be an outcome of reverberation in the PFC-MDN circuit (Alexander and Fuster, 1973). In monkeys, consistent with this idea, similar proportions of DLPFC and MDN neurons showed sustained activity during delay period with similar discharge characteristics (Fuster and Alexander, 1971, 1973; Tanibuchi and Goldman-Rakic, 2003; Watanabe and Funahashi, 2004a). Moreover, inactivation of the DLPFC disrupted sustained elevation of delay-period neural activity in the MDN (Alexander and Fuster, 1973). However, in rats, we found only a small fraction of neurons that conveyed retrospective/prospective information throughout delay period (continuous differential delay cells) in the mPFC (Baeg et al., 2003) as well as MDN (present results). Hence, it is unlikely that working memory is mediated by sustained elevation of delay-period neural activity based on PFC-MDN reverberation in rats. PFC-MDN interactions might still support working memory based on sequential activation of different groups of cells (Miller, 1996; Baeg et al., 2003; Fujisawa et al., 2008) rather than sustained activity of individual neurons during delay period, however.

Even in monkeys, it is unclear whether and how MDN-PFC interactions contribute to working memory. Funahashi and colleagues have shown that the content of working memory is transformed from retrospective (cue-related) to prospective (response-related) memory much earlier in the MDN than in the DLPFC (Takeda and Funahashi, 2004; Watanabe et al., 2009; Watanabe and Funahashi, 2012). These results suggest that simple reverberation for static maintenance of sensory information is an unlikely scenario for PFC-MDN interactions. Rather, the DLPFC and MDN are likely to undergo dynamic interactions during delay period, which remain to be explored in future studies. It should be noted that our results do not exclude potential roles of other types of PFC-MDN interactions in mediating working memory in rats. For example, phasic MDN inputs to the PFC might trigger updating of the content of working memory maintained in the PFC (Hazy et al., 2006).

Our results lead to a potential puzzle that needs to be resolved. On the one hand, numerous lesion/inactivation studies have shown requirement of intact MDN for correctly performing a working memory task in rats (e.g., Winocur, 1985; Stokes and Best, 1990; Harrison and Mair, 1996; Floresco et al., 1999; Romanides et al., 1999). On the other hand, our results show that the MDN does not maintain retrospective/prospective information required to perform a working memory task. What role, then, does the MDN play during a working memory task? The MDN has been implicated in stimulus-reward and response-reward associations (Oyoshi et al., 1996; Chudasama et al., 2001; Corbit et al., 2003; Kawagoe et al., 2007; Yu et al., 2012). MDN lesion/inactivation might have disrupted its role in stimulus-reward and/or response-reward association, thereby impairing performance in a working memory task. In line with this possibility, we found that MDN neurons concurrently encoded reward (i.e., choice outcome) and goal location, which might reflect its function in stimulus-reward or response-reward association. Alternatively, the rodent MDN might play an essential role in working memory by other means than providing persistent retrospective/prospective information, such as providing trigger signals to switch the content of working memory in the PFC (Hazy et al., 2006). Additional studies are needed to resolve this matter.

The possibility that MDN may be involved in response-reward associations fits well with our findings that delay-period MDN neural activity conveyed more information about the previous than upcoming goal choice, especially during early delay period. This finding is consistent with the results of previous physiological studies in rats. In a Pavlovian conditioning task, some MDN neurons fired differently in response to reward- vs. punishment-predicting cues during the delay period between conditioned and unconditioned stimuli. Because the animal's response (as monitored by electromyograms) was evident only during the late phase of the delay period, this finding suggests that some MDN neurons conveyed retrospective sensory information at least during the early delay period (Oyoshi et al., 1996). We also have found that several brain structures anatomically related to the MDN, such as the mPFC, orbitofrontal cortex, secondary motor cortex, dorsal striatum, ventral striatum, and hippocampus, carry previous choice signals that gradually decay over time during a dynamic foraging task (Kim et al., 2009, 2013; Sul et al., 2010, 2011; Lee et al., 2012). Neural activity related to past actions found in the MDN might be a general characteristic for those brain structures involved in response-reward association; it might serve a role of eligibility trace that can bridge a temporal gap between an action and its outcome (temporal credit assignment problem; Sutton and Barto, 1998).

It is unclear whether the difference in behavioral task or animal species is responsible for the different outcomes of the present and previous monkey studies (Fuster and Alexander, 1971, 1973; Tanibuchi and Goldman-Rakic, 2003; Watanabe and Funahashi, 2004a,b). Many aspects of the behavioral tasks employed in the present and previous monkey studies are different. In particular, rats were required to navigate to different locations in our task, whereas monkeys stayed at one location with their heads fixed in previous monkey studies. Differences in behavioral task might demand the engagement of the MDN in the maintenance of working memory to different degrees. Another possibility is that neural mechanisms for maintaining working memory are different between rodents and monkeys. Of various cortical regions, the PFC is particularly well developed in primates compared to other mammals. Currently, the homology between rodent and primate PFC is unclear. In particular, it has been controversial whether there exists a rodent homolog of primate DLPFC (Preuss, 1995; Wise, 2008), although rodent mPFC has been proposed to be the one (Kolb, 1990; Uylings et al., 2003; Vertes, 2006). Thus, it is possible that neural systems and underlying processes supporting working memory might differ between the rodent and primate brain.

### Conflict of interest statement

The authors declare that the research was conducted in the absence of any commercial or financial relationships that could be construed as a potential conflict of interest.
